# The genome sequence of the common green furrow bee, *Lasioglossum morio *(Fabricius, 1793)

**DOI:** 10.12688/wellcomeopenres.18715.1

**Published:** 2023-01-18

**Authors:** Steven Falk, University of Oxford and Wytham Woods Genome Acquisition Lab, Darwin Tree of Life Barcoding collective, Wellcome Sanger Institute Tree of Life programme, Wellcome Sanger Institute Scientific Operations: DNA Pipelines collective, Tree of Life Core Informatics collective, Joseph Monks, Darwin Tree of Life Consortium

**Affiliations:** 1 Independent Researcher, Kenilworth, Warwickshire, UK; 2 Department of Life Sciences- Hymenoptera section, Natural History Museum, London, UK

**Keywords:** Lasioglossum morio, common green furrow bee, genome sequence, chromosomal, Hymenoptera

## Abstract

We present a genome assembly from an individual male *Lasioglossum morio* (the common green furrow bee; Arthropoda; Insecta; Hymenoptera; Halictidae). The genome sequence is 547 megabases in span. Over half of the assembly (55.79%) is scaffolded into 12 chromosomal pseudomolecules. The mitochondrial genome was also assembled, and is 16.8 kilobases in length. Gene annotation of this assembly on Ensembl identified 11,460 protein coding genes.

## Species taxonomy

Eukaryota; Metazoa; Ecdysozoa; Arthropoda; Hexapoda; Insecta; Pterygota; Neoptera; Endopterygota; Hymenoptera; Apocrita; Aculeata; Apoidea; Halictidae; Halictinae; Halictini; *Lasioglossum*; *Dialictus*; *Lasioglossum morio* (Fabricius, 1793) (NCBI:txid88514).

## Background

*Lasioglossum (Dialictus) morio* (green furrow bee) is one of four small (forewing length of 3.5–4.5 mm) species in the UK belonging to the subgenus *Dialictus*, characterised by their metallic green bodies. The species can be distinguished from the three other UK species of this subgenus by the shape and density of the punctures on the scutum ( Falk, 2015). In Great Britain, the species is widespread throughout England and Wales, but no records exist from Scotland and Ireland ( Else, 2005). In southern Britain and the Channel Islands it is usually a very common bee, colonising patches of exposed soil as nesting sites.

*Lasioglossum morio* is polylectic but shows a particular preference for sallows and *Prunus spinosa* in the spring. The species can be particularly abundant in urban environments ( Fischer *et al.*, 2016), and it can be found nesting in *soft mortar walls.* Like many other halictid species, *L. morio* is primitively eusocial and nests occur in large aggregations on south-facing slopes ( Falk, 2015). Females emerge in March with males appearing in June. Both sexes are active until October ( Else, 2005). *L. morio is a host of the cleptoparastic bee Sphecodes niger and possibly S. geoffrellus* and *Nomada sheppardana;* also the conopid fly *Thecophora atra.*

The genome of the common green furrow bee, *L. morio,* was sequenced as part of the Darwin Tree of Life Project, a collaborative effort to sequence all named eukaryotic species in the Atlantic Archipelago of Britain and Ireland. By sequencing the genome of *L. morio* we hope to both offer additional data that may form the foundations of a toolkit for the deployment of eDNA and other approaches to monitoring otherwise inaccessible biodiversity.

## Genome sequence report

**Figure 1.**
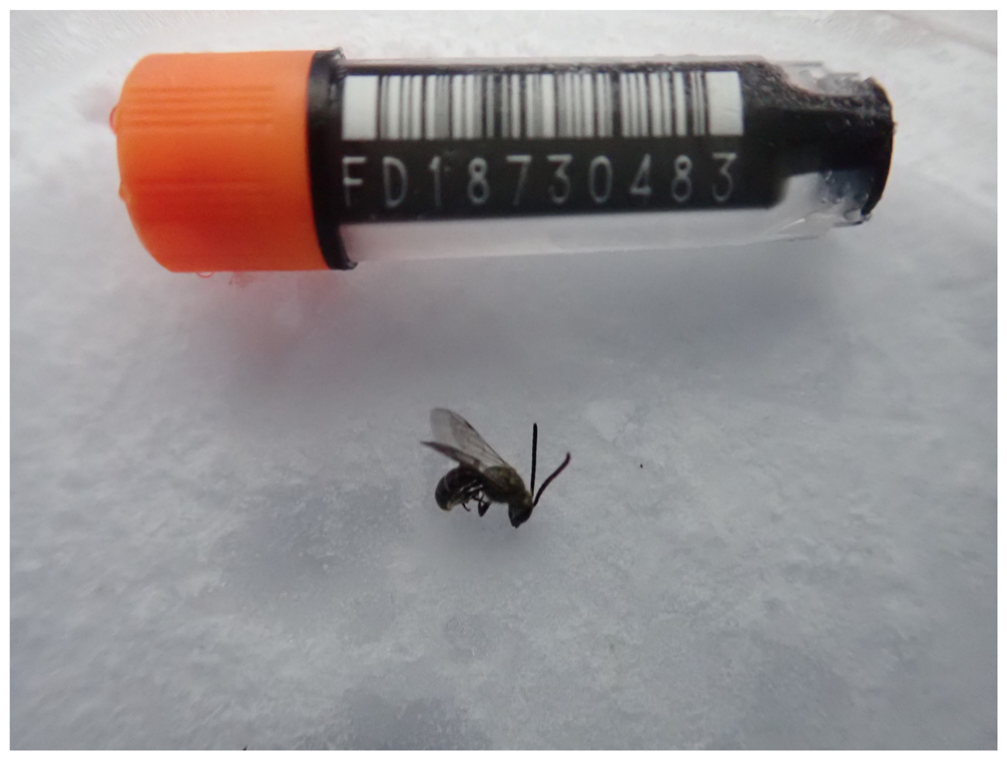
Image of the *Lasioglossum morio* specimen (iyLasMori1) taken during preservation and processing.

The genome was sequenced from a single male *L. morio* specimen (iyLasMori1) ( Figure 1) collected from Wytham Woods, Oxfordshire (biological vice-county: Berkshire), UK (latitude 51.77, longitude –1.309). A total of 35-fold coverage in Pacific Biosciences single-molecule long reads and 51-fold coverage in 10X Genomics read clouds was generated. Primary assembly contigs were scaffolded with chromosome conformation Hi-C data, derived from a second male specimen, iyLasMori2. Manual assembly curation corrected 66 missing joins and misjoins, increasing the assembly size by 8.21%, the scaffold number by 89.55% and the scaffold N50 by 25.84%.

**Figure 2.**
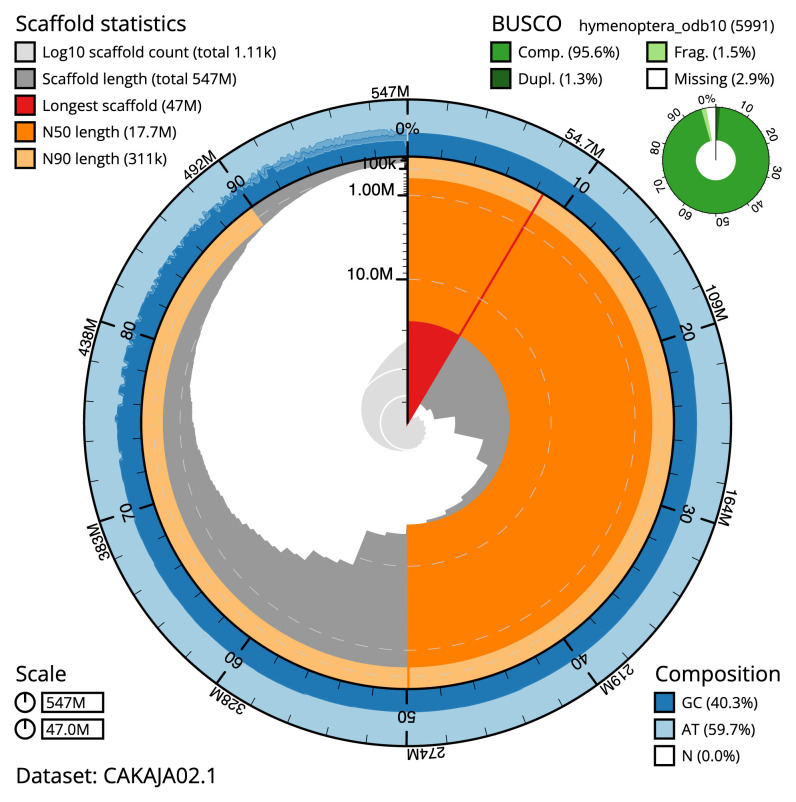
Genome assembly of *Lasioglossum morio*, iyLasMori1.2: metrics. The BlobToolKit Snailplot shows N50 metrics and BUSCO gene completeness. The main plot is divided into 1,000 size-ordered bins around the circumference with each bin representing 0.1% of the 478,951,010 bp assembly. The distribution of chromosome lengths is shown in dark grey with the plot radius scaled to the longest chromosome present in the assembly (51,674,092 bp, shown in red). Orange and pale-orange arcs show the N50 and N90 chromosome lengths (27,713,800 and 136,982 bp), respectively. The pale grey spiral shows the cumulative chromosome count on a log scale with white scale lines showing successive orders of magnitude. The blue and pale-blue area around the outside of the plot shows the distribution of GC, AT and N percentages in the same bins as the inner plot. A summary of complete, fragmented, duplicated and missing BUSCO genes in the hymenoptera_odb10 set is shown in the top right. An interactive version of this figure is available at https://blobtoolkit.genomehubs.org/view/iyLasMori1.2/dataset/CAKAJA02.1/snail.

The final assembly has a total length of 547 Mb in 1,1107 sequence scaffolds with a scaffold N50 of 17.7 Mb ( Table 1). Of the assembly sequence, 55.79% was assigned to 12 chromosomal-level scaffolds confirmed by the Hi-C data, which are named in order of size ( Figure 2– Figure 5; Table 2). Several repeat types can be seen to cluster by Hi-C yet have no association with the defined chromosomes. These repeats make up a large proportion of the assembly ( Figure 5). The assembly has a BUSCO v5.2.2 ( Manni *et al.*, 2021) completeness of 95.7% (single 94.4%, duplicated 1.3%) using the hymenoptera_odb10 reference set ( *n* = 5,991).

**Table 1.** Genome data for *Lasioglossum morio*, iyLasMori1.2.

*Project accession data*	
Assembly identifier	iyLasMori1.2.
Species	*Lasioglossum morio*
Specimen	iyLasMori1
NCBI taxonomy ID	88514
BioProject	PRJEB46298
BioSample ID	SAMEA7746456
Isolate information	iyLasMori1 (DNA sequencing), iyLasMori2 (HiC sequencing)
*Genome assembly*	
Assembly accession	GCA_916610235.2
Span (Mb)	479
Number of contigs	1430
Contig N50 length (Mb)	4.1
Number of scaffolds	1143
Scaffold N50 length (Mb)	27.7
Longest scaffold (Mb)	47
Assembly metrics *	
Base pair QV	51.6 (Benchmark: ≥50)
*k*-mer completeness	99.96% (Benchmark: ≥95%)
BUSCO **	C:95.7%[S:94.4%,D:1.3%],F:1.5%,M:2.9%, n:5,991 (Benchmark: C ≥ 95%)
Percentage of assembly mapped to chromosomes	55.79% (Benchmark: ≥95%)
Organelles	Mitochondrial genome (Benchmark: complete single alleles)
*Raw data accessions*	
PacificBiosciences SEQUEL II	ERR6939225
10X Genomics Illumina	ERR6688416–ERR6688419
Hi-C Illumina	ERR6688415
Genome annotation	
Number of protein-coding genes	11,460

* Assembly metric benchmarks are adapted from column VGP-2020 of “Table 1: Proposed standards and metrics for defining genome assembly quality” from ( Rhie *et al.*, 2021). ** BUSCO scores based on the hymenoptera_odb10 BUSCO set using v5.2.2. C = complete [S = single copy, D = duplicated], F = fragmented, M = missing, n = number of orthologues in comparison. A full set of BUSCO scores is available at https://blobtoolkit.genomehubs.org/view/iyLasMori1.2/dataset/CAKAJA02.1/busco.

**Figure 3.**
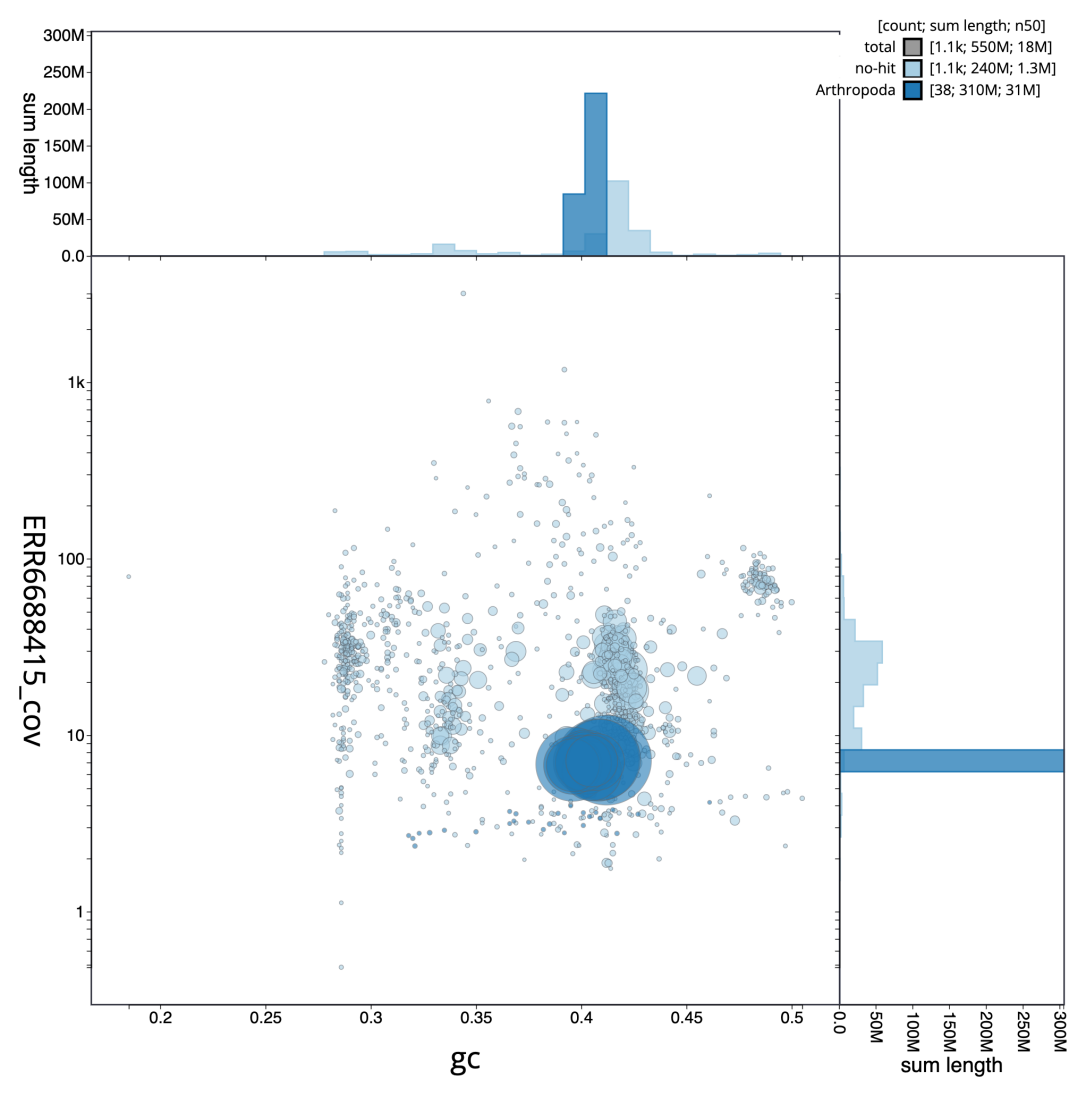
Genome assembly of *Lasioglossum morio*, iyLasMori1.2: GC coverage. BlobToolKit GC-coverage plot. Scaffolds are coloured by phylum. Circles are sized in proportion to scaffold length. Histograms show the distribution of scaffold length sum along each axis. An interactive version of this figure is available at https://blobtoolkit.genomehubs.org/view/iyLasMori1.2/dataset/CAKAJA02.1/blob.

**Figure 4.**
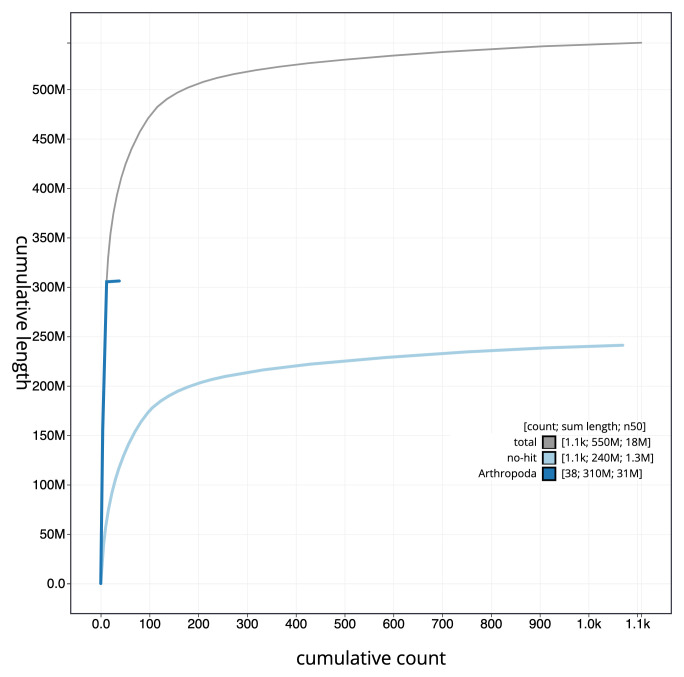
Genome assembly of *Lasioglossum morio*, iyLasMori1.2: cumulative sequence. BlobToolKit cumulative sequence plot. The grey line shows cumulative length for all scaffolds. Coloured lines show cumulative lengths of scaffolds assigned to each phylum using the buscogenes taxrule. An interactive version of this figure is available at https://blobtoolkit.genomehubs.org/view/iyLasMori1.2/dataset/CAKAJA02.1/cumulative.

**Figure 5.**
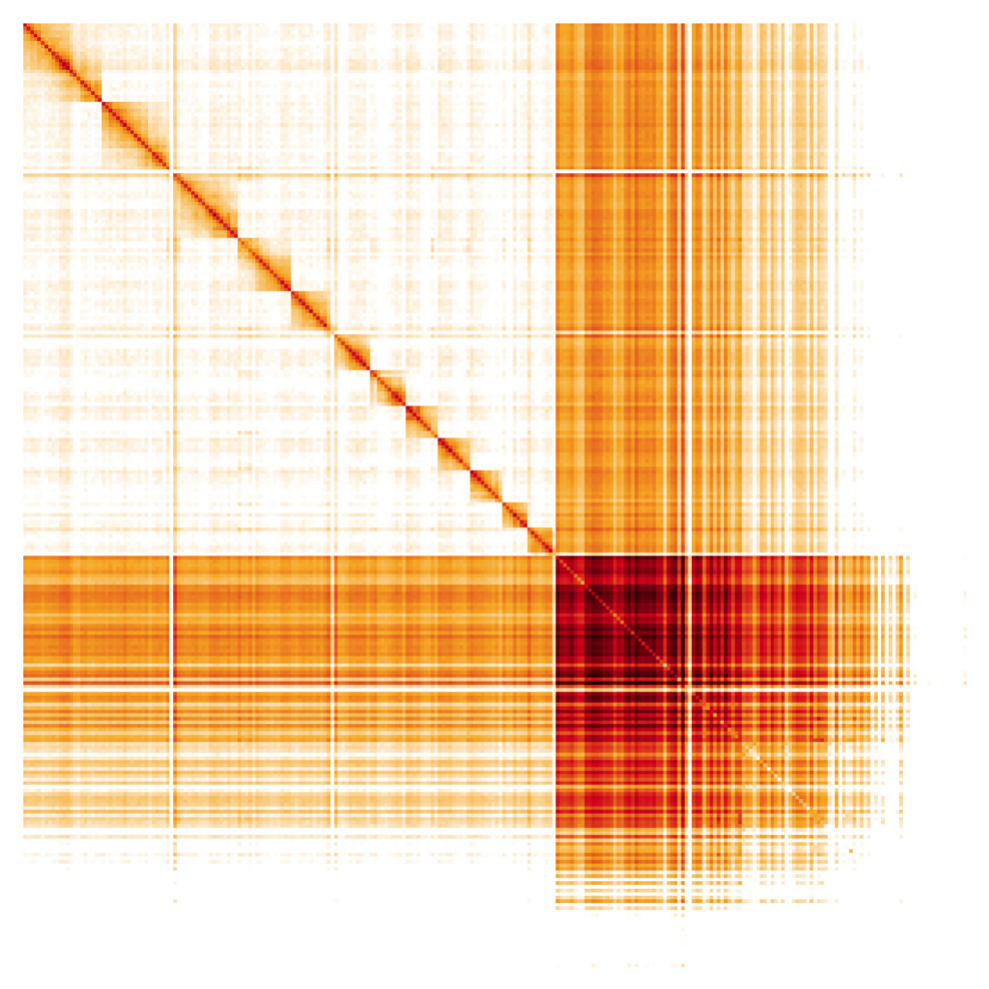
Genome assembly of *Lasioglossum morio*, iyLasMori1.2: Hi-C contact map. Hi-C contact map of the iyLasMori1.2 assembly, visualised in HiGlass. Chromosomes are shown in size order from left to right and top to bottom. An interactive version of this figure is available at https://genome-note-higlass.tol.sanger.ac.uk/l/?d=BCxE-IaES7KjadU8Sq2eHA.

**Table 2.** Chromosomal pseudomolecules in the genome assembly of *Lasioglossum morio, iyLasMori1.2*.

INSDC accession	Name	Size (Mb)	GC%
OU744323.1	1	46.98	41.2
OU744324.1	2	39.12	40.8
OU744325.1	3	37.81	40.9
OU744326.1	4	31.47	39.6
OU744327.1	5	23.46	40.2
OU744328.1	6	20.26	40.2
OU744329.1	7	20.1	40.5
OU744330.1	8	19.24	39.6
OU744331.1	9	18.38	40.7
OU744332.1	10	17.69	40.1
OU744333.1	11	15.77	39.6
OU744334.1	12	15.02	40.5
OU744335.1	MT	0.02	18.5
-	-	241.88	40.1

## Genome annotation report

The iyLasMori1.2 (GCA_916610235.2) genome was annotated using the Ensembl rapid annotation pipeline ( Table 1; https://rapid.ensembl.org/Lasioglossum_morio_GCA_916610235.2/Info/Index). The resulting annotation includes 27,469 transcribed mRNAs from 11,460 protein-coding and 4,137 non-coding genes.

## Methods

### Sample acquisition and DNA extraction

Two male *L. morio* specimens (iyLasMori1 and iyLasMori2) specimens were caught using a net in Wytham Woods, Oxfordshire (biological vice-county: Berkshire), UK (latitude 51.77, longitude –1.309) by Steven Falk, Independent Researcher. The samples were identified by Steven Falk and snap-frozen on dry ice.

DNA was extracted at the Tree of Life laboratory, Wellcome Sanger Institute. The iyLasMori1 sample was weighed and dissected on dry ice with tissue set aside for Hi-C sequencing. Whole organism tissue was disrupted using a Nippi Powermasher fitted with a BioMasher pestle. Fragment size analysis of 0.01–0.5 ng of DNA was then performed using an Agilent FemtoPulse. High molecular weight (HMW) DNA was extracted using the Qiagen MagAttract HMW DNA extraction kit. Low molecular weight DNA was removed from a 200 ng aliquot of extracted DNA using 0.8X AMpure XP purification kit prior to 10X Chromium sequencing; a minimum of 50 ng DNA was submitted for 10X sequencing. HMW DNA was sheared into an average fragment size of 12–20 kb in a Megaruptor 3 system with speed setting 30. Sheared DNA was purified by solid-phase reversible immobilisation using AMPure PB beads with a 1.8X ratio of beads to sample to remove the shorter fragments and concentrate the DNA sample. The concentration of the sheared and purified DNA was assessed using a Nanodrop spectrophotometer and Qubit Fluorometer and Qubit dsDNA High Sensitivity Assay kit. Fragment size distribution was evaluated by running the sample on the FemtoPulse system.

### Sequencing

Pacific Biosciences HiFi circular consensus and 10X Genomics read cloud sequencing libraries were constructed according to the manufacturers’ instructions. Sequencing was performed by the Scientific Operations core at the Wellcome Sanger Institute on Pacific Biosciences SEQUEL II and Illumina NovaSeq 6000 instruments. Hi-C data were generated from whole organism tissue of iyLasMori2 using the Arima v2.0 kit and sequenced on an Illumina NovaSeq 6000 instrument.

### Genome assembly

Assembly was carried out with Hifiasm ( Cheng *et al.*, 2021). Haplotypic duplication was identified and removed with purge_dups ( Guan *et al.*, 2020). Scaffolding with Hi-C data ( Rao *et al.*, 2014) was carried out with SALSA2 ( Ghurye *et al.*, 2019). The Hi-C scaffolded assembly was polished with the 10X Genomics Illumina data by aligning to the assembly with Long ranger ALIGN, calling variants with freebayes ( Garrison & Marth, 2012). The mitochondrial genome was assembled with MitoHiFi ( Uliano-Silva *et al.*, 2021), which performed annotation using MitoFinder ( Allio *et al.*, 2020). The assembly was checked for contamination as described previously ( Howe *et al.*, 2021). Manual curation ( Howe *et al.*, 2021) was performed using HiGlass ( Kerpedjiev *et al.*, 2018) and Pretext ( Harry, 2022). The genome was analysed within the BlobToolKit environment ( Challis *et al.*, 2020). Table 3 contains a list of all software tool versions used, where appropriate.

**Table 3.** Software tools used.

Software tool	Version	Source
BlobToolKit	3.0.5	Challis *et al.*, 2020
freebayes	1.3.1-17-gaa2ace8	Garrison & Marth, 2012
Hifiasm	0.15.2	Cheng *et al.*, 2021
HiGlass	1.11.6	Kerpedjiev *et al.*, 2018
Long ranger ALIGN	2.2.2	https://support.10xgenomics.com/genome- exome/software/pipelines/latest/advanced/ other-pipelines
MitoHiFi	2.0	Uliano-Silva *et al.*, 2021
PretextView	0.2.x	Harry, 2022
purge_dups	1.2.3	Guan *et al*., 2020
SALSA2	2.2	Ghurye *et al.*, 2019

### Genome annotation

The Ensembl gene annotation system ( Aken *et al.*, 2016) was used to generate annotation for the *L. morio* assembly (GCA_916610235.2). Annotation was created primarily through alignment of transcriptomic data to the genome, with gap filling via protein to-genome alignments of a select set of proteins from UniProt ( UniProt Consortium, 2019).

### Ethics/compliance issues

The materials that have contributed to this genome note have been supplied by a Darwin Tree of Life Partner. The submission of materials by a Darwin Tree of Life Partner is subject to the Darwin Tree of Life Project Sampling Code of Practice. By agreeing with and signing up to the Sampling Code of Practice, the Darwin Tree of Life Partner agrees they will meet the legal and ethical requirements and standards set out within this document in respect of all samples acquired for, and supplied to, the Darwin Tree of Life Project. Each transfer of samples is further undertaken according to a Research Collaboration Agreement or Material Transfer Agreement entered into by the Darwin Tree of Life Partner, Genome Research Limited (operating as the Wellcome Sanger Institute), and in some circumstances other Darwin Tree of Life collaborators.

## Data Availability

European Nucleotide Archive: *Lasioglossum morio* (common green furrow bee). Accession number PRJEB46298; https://identifiers.org/ena.embl/PRJEB46298 ( Wellcome Sanger Institute, 2023). The genome sequence is released openly for reuse. The *L. morio* genome sequencing initiative is part of the Darwin Tree of Life (DToL) project. All raw sequence data and the assembly have been deposited in INSDC databases.

## Author information

Members of the University of Oxford and Wytham Woods Genome Acquisition Lab are listed here: https://doi.org/10.5281/zenodo.4789928.

Members of the Darwin Tree of Life Barcoding collective are listed here: https://doi.org/10.5281/zenodo.4893703.

Members of the Wellcome Sanger Institute Tree of Life programme are listed here: https://doi.org/10.5281/zenodo.4783585.

Members of Wellcome Sanger Institute Scientific Operations: DNA Pipelines collective are listed here: https://doi.org/10.5281/zenodo.4790455.

Members of the Tree of Life Core Informatics collective are listed here: https://doi.org/10.5281/zenodo.5013541.

Members of the Darwin Tree of Life Consortium are listed here: https://doi.org/10.5281/zenodo.4783558.
