# Supplementary material for: The genome sequence of the common green furrow bee, Lasioglossum morio (Fabricius, 1793)
Source: Wellcome Open Res. 2024 Mar 25;8:28. Originally published 2023 Jan 18. [Version 2] doi: 10.12688/wellcomeopenres.18715.2 (PMC11063680; doi:10.12688/wellcomeopenres.18715.2)
Supplement: Peer review history

# Reviewer report: Reviewer response for version 2

**DOI:** 10.21956/wellcomeopenres.23543.r77638

**Published:** 2024-05-01

**Authors:** Miriam Richards

**Affiliations:** 1 Brock University, Saint Catharines, Ontario, Canada

1. *soft mortar walls - remove italics*
2. Please confirm the number of scaffolds. Currently the text reads 'The final assembly has a total length of 547 Mb in 1,1107 sequence scaffolds...' - possibly a misplaced comma?

Are sufficient details of methods and materials provided to allow replication by others?

Yes

Is the rationale for creating the dataset(s) clearly described?

Yes

Are the datasets clearly presented in a useable and accessible format?

Partly

Are the protocols appropriate and is the work technically sound?

Yes

Reviewer Expertise:

Social evolution and ecology of sweat bees and carpenter bees

I confirm that I have read this submission and believe that I have an appropriate level of expertise to confirm that it is of an acceptable scientific standard.

# Reviewer report: Reviewer response for version 2

**DOI:** 10.21956/wellcomeopenres.23543.r77637

**Published:** 2024-03-27

**Authors:** Pilar De la Rúa

**Affiliations:** 1 Department of Zoology and Physical Anthropology, Faculty of Veterinary, University of Murcia, Murcia, Spain

No further comments

Are sufficient details of methods and materials provided to allow replication by others?

Yes

Is the rationale for creating the dataset(s) clearly described?

Yes

Are the datasets clearly presented in a useable and accessible format?

Yes

Are the protocols appropriate and is the work technically sound?

Yes

Reviewer Expertise:

pollinators population genetics and conservation

I confirm that I have read this submission and believe that I have an appropriate level of expertise to confirm that it is of an acceptable scientific standard.

# Reviewer report: Reviewer response for version 1

**DOI:** 10.21956/wellcomeopenres.20753.r57797

**Published:** 2023-06-15

**Authors:** Pilar De la Rúa

**Affiliations:** 1 Department of Zoology and Physical Anthropology, Faculty of Veterinary, University of Murcia, Murcia, Spain

This manuscript presents the details of the process of obtaining the genome assembly of a small bee in order to obtain a squence baselint for further studies.

 The work is straightforward and the whole process is well detailed. I have only a few editing notes to add:

 - In the background section, second paragraph some words are in italics. Please recheck this

Are sufficient details of methods and materials provided to allow replication by others?

Yes

Is the rationale for creating the dataset(s) clearly described?

Yes

Are the datasets clearly presented in a useable and accessible format?

Yes

Are the protocols appropriate and is the work technically sound?

Yes

Reviewer Expertise:

pollinators population genetics and conservation

I confirm that I have read this submission and believe that I have an appropriate level of expertise to confirm that it is of an acceptable scientific standard.

# Reviewer report: Reviewer response for version 1

**DOI:** 10.21956/wellcomeopenres.20753.r57060

**Published:** 2023-05-26

**Authors:** Miriam Richards

**Affiliations:** 1 Brock University, Saint Catharines, Ontario, Canada

This paper provides a chromosome-level genome sequence for the eusocial sweat bee, *Lasioglossum (Dialictus) morio*. The genome was sequenced from a single male specimen collected from the UK. The paper is a basic description of genome characteristics, including comments on sequencing quality and completeness, genome size, and putative numbers of chromosomes and protein-coding sequences. The data were filtered to ascertain as far as possible, that the inferred sequences were indeed for this species, and not for possible parasites or symbionts. 

 The justification for publishing the genome of this species is simple, in my opinion – for the same reasons that unknown species should be named and described, so too should their genomes. It was rather refreshing to read this paper because the genome is being published without an ad hoc justification or theoretical context justifying the publication of the genome of this particular species. I think we are still at the stage where accumulating accurate genome sequences is in and of itself a useful scientific enterprise, and I am happy to see this new bee genome. 

 That being said, I have two concerns with this paper:

1. First, sweat bees in the subgenus *Dialictus* are notoriously difficult to identify.  While the first author of this paper is undoubtedly expert at bee identification, I am surprised that there is no mention of voucher specimens being deposited for future reference. If no formal key is available, is it possible to provide a description of key taxonomic characteristics that strengthen confidence that the specimen was indeed correctly named?
2. Second, I found the minimalist presentation of the bioinformatics methods was bare bones as to be confusing. Given the number of available software packages and pipelines for assembling genomes, and given that these may differ in ways subtle and not so subtle, I think it would be helpful to readers of the authors to 1) provide some context for the software chosen and 2) provide some idea of how their software choices may have affected their characterization of the genome.  
  Another reason to provide a little more clarity for the bioinformatics methods is that the figures provided are likely to be difficult for readers to interpret unless they are familiar with the software used to generate them (BlobToolKit, HiGlass). For example, Figure 5 is cited as visual evidence of a high degree of repetitive sequences in this *L. morio* genome – but the figure has no labels and so this reader, at least, was left to guess at what it means (the interactive version of this figure also provides no information except for a colour scale without units).

Are sufficient details of methods and materials provided to allow replication by others?

Yes

Is the rationale for creating the dataset(s) clearly described?

Yes

Are the datasets clearly presented in a useable and accessible format?

Partly

Are the protocols appropriate and is the work technically sound?

Yes

Reviewer Expertise:

Social evolution and ecology of sweat bees and carpenter bees

I confirm that I have read this submission and believe that I have an appropriate level of expertise to confirm that it is of an acceptable scientific standard, however I have significant reservations, as outlined above.

# Response

**Published:** 2024-03-15

**Authors:** Tree of Life Team Sanger

**Affiliations:** Wellcome Trust Sanger Institute, UK

Thank you for your comments. In response to the comment "is it possible to provide a description of key taxonomic characteristics that strengthen confidence that the specimen was indeed correctly named": The specimen was identified at collection using a species key, and this identification was confirmed during the assembly process by performing a COI barcode check, which is a standard part of the pipeline. We have included a link to the TOLQC page in the article, which provides data about the assembly and also the BOLD barcode check. 

 In response to the comment that the figures are difficult to interpret: We have inserted an explanation about Figures 2 to 4 in the Genome sequence report section. We have added a chromosome grid to Figure 5, so that readers can see the repeated types associated with Hi-C that do not map to the chromosomes.

 We hope that these changes meet with your approval.
